# Molecular Targets for Foodborne Pathogenic Bacteria Detection

**DOI:** 10.3390/pathogens12010104

**Published:** 2023-01-08

**Authors:** Spiros Paramithiotis

**Affiliations:** Laboratory of Food Process Engineering, Department of Food Science and Human Nutrition, Agricultural University of Athens, 75 Iera Odos St., 11855 Athens, Greece; sdp@aua.gr

**Keywords:** antibodies, aptamers, lectins, metabolites, nucleic acids, *Listeria monocytogenes*, *Salmonella*, *Escherichia coli*

## Abstract

The detection of foodborne pathogenic bacteria currently relies on their ability to grow on chemically defined liquid and solid media, which is the essence of the classical microbiological approach. Such procedures are time-consuming and the quality of the result is affected by the selectivity of the media employed. Several alternative strategies based on the detection of molecular markers have been proposed. These markers may be cell constituents, may reside on the cell envelope or may be specific metabolites. Each marker provides specific advantages and, at the same time, suffers from specific limitations. The food matrix and chemical composition, as well as the accompanying microbiota, may also severely compromise detection. The aim of the present review article is to present and critically discuss all available information regarding the molecular targets that have been employed as markers for the detection of foodborne pathogens. Their strengths and limitations, as well as the proposed alleviation strategies, are presented, with particular emphasis on their applicability in real food systems and the challenges that are yet to be effectively addressed.

## 1. Introduction

Each year, hundreds of thousands of people are infected, thousands are hospitalized, and many die from some foodborne disease. In the United States, as many as 9 million people are getting sick each year, 56,000 are hospitalized, and 1,300 die [1]. In the European Union, a total of 186,000 confirmed cases were reported in 2020, resulting in 17,000 hospitalizations and 330 deaths [2]. These numbers indicate that despite the implementation of quality control systems, there is still room for improvement, at least regarding monitoring and control of *Salmonella*, shiga toxin-producing *Escherichia coli*, *Campylobacter* spp. and *Listeria monocytogenes*, to which the majority of foodborne illnesses have been attributed.

The fast and accurate detection of foodborne pathogens has been a standing quest for food industries and government agencies. Therefore, this subject has been extensively studied; every relevant technological and methodological advancement is also evaluated from a food safety assessment perspective. However, the classical microbiological approach still remains the golden standard. Classical microbiological protocols rely on the detection of the pathogen itself, which occurs after the incubation of a test portion under optimal conditions for each pathogen. The growth substrate is supplemented with compounds, such as antibiotics, that suppress the growth of other microorganisms, including the native microbiota of the commodity under examination. The presence of a pathogenic microorganism can be declared only after phenotypical verification of its identity. The major disadvantage of this approach is the time-consuming nature of the procedure. For example, the detection of *L. monocytogenes* according to the ISO 11290-1 procedure [3] may require up to nine days, which exceeds the shelf life of several commodities, such as fresh salads.

Several alternative strategies that rely on the detection of a molecular target have been developed and proposed. The principal aim of such assays is to shorten the time required for detection without affecting specificity and sensitivity. In addition, they may lead to the development of biosensors, i.e., devices designed to automatically carry out detection and/or quantification [4] and therefore operatable by technicians. Depending on the localization of the target, three approaches may be distinguished: (i) detection of molecular targets that reside on the surface of the bacterial cells leading to the selective capturing of the cells of the pathogen, (ii) detection of cellular components that only occur within the cells of the pathogen under surveillance and (iii) detection of metabolites that are produced by the pathogen during its growth or subsistence in a specific commodity. In all cases, application in real food samples is challenged by: (i) the presence of a native background microbiota, the population of which is usually several orders of magnitude larger than the population of the pathogen under surveillance, (ii) the chemical composition of the food, and (iii) strain diversity, referring to the pathogen under surveillance. These, irrespective of the adopted detection approach, may increase the number of false positive results due to the detection of non-specific targets. In addition, they may also increase the number of false negative results due to inhibition of the desired interactions or, especially in the case of strain diversity, the absence or the modification of the target molecule. These issues are addressed with the incorporation of pretreatment steps prior to the detection step. Selective enrichment is, in most cases, the employed pretreatment since it allows increase of the target pathogen population, decrease of the population of the background microbiota and dilution of the inhibitory substances. Strain diversity can only be addressed through the selection of targets that are present in all strains of the pathogen under surveillance. In addition, each detection approach is characterized by specific advantages and limitations. In all cases, sensitivity and specificity heavily rely on the selected molecular target and are affected by the analytical steps employed for visualization of the detection.

Several review articles are published every year, summarizing the advancements in biosensor development, focusing mostly on the visualization step. However, little attention is given to the molecular targets themselves, as well as the strategies that can be used for the improvement of detection sensitivity and specificity. Therefore, the aim of the present article is to collect all available information regarding the nature of molecular targets that have been employed in foodborne pathogenic bacteria detection and their capturing strategies, as well as to critically evaluate and discuss their utilization and applicability in actual food systems.

## 2. Surface-Residing Molecular Targets

The bacterial cell envelope plays an important role in cell integrity and function since it regulates interactions with the environment. On the basis of the basic structure and organization of the bacterial cell envelope, bacterial cells have been distinguished into Gram positive and negative. In both cases, the cell envelope structure and function present inter- and intra-species differences due to their adaptation to specific habitats. The outer surface of the bacterial envelope contains a variety of proteins, the function of which is associated with the microenvironment and is therefore subjected to qualitative and quantitative changes according to environmental stimuli [5]. These proteins have been extensively used as molecular targets for the detection of foodborne pathogens. An ideal target should fulfil the following two criteria: (i) it should be expressed under the conditions employed for the detection by all strains of the pathogen and not by other bacteria that may also participate in the microecosystem under examination, and (ii) it should be strongly associated with the surface of the pathogen [6]. Such is the case of *L. monocytogenes* and its cell envelope-associated proteins, such as InlA and InlB. Both proteins have a very important role in pathogenicity, as they mediate entry into epithelial cells [7,8]. InlA contains an LPXTG sequence motif that allows covalent linkage to the cell wall. In contrast, InlB does not contain such a motif, but anchoring to the cell wall is mediated by the carboxy terminal amino acids [7]. The antigenic potential of these proteins has led to the development of antibodies and concomitant immunological approaches for the detection of the pathogen [9,10]. In addition, the antigenic potential of other surface proteins, such as *Listeria* adhesion protein B (LapB), which is an LPXTG protein associated with the entry of the pathogen into eukaryotic cells, and surface autolysin (IspC), which is an autolysin possessing N-acetylglycosaminidase activity, have also been exhibited [11,12]. The antigenic potential of secreted proteins, such as listeriolysin and phosphatidylinositol-specific phospholipase C (PI-PLC), both of which are necessary for the escape of the pathogen from the phagocytic vacuoles of the host, has also been employed for antibody development. In the case of listeriolysin, quite promising immunological approaches for listeriosis diagnosis have been developed [13,14]. In contrast, the use of PI-PLC resulted in extended cross-reactivity due to its conserved nature among listeriae and non-*Listeria* genera, such as *Bacillus* spp. and *Clostridium* spp. [14].

Apart from the proteins, the antigenic potential of the lipopolysaccharide (LPS) layer, which resides in the outer membrane of the cell envelope of Gram-negative bacteria, has also been considered. More specifically, the majority of assays developed for *Salmonella* target the O-antigenic polysaccharide chain in the LPS layer [15]. Only a few exceptions have been reported, namely the detection of flagella antigens [16,17] and PagC, an outer membrane protein belonging to the porin superfamily, antibodies [18]. In the case of flagella antigens, cross-reactivity between *Salmonella* serovars as well as with other Gram-negative pathogenic bacteria, such as *Campylobacter coli*, *C. jejuni*, *E. coli*, *Helicobacter pylori* and *Yersinia enterocolitica*, has been reported [16,17]. On the other hand, the detection of PagC antibodies seems to be a promising alternative [18].

In the next paragraphs, capturing of surface-residing molecular targets by antibodies, aptamers and lectins as well as the development of assays for the detection of foodborne pathogens in actual food samples is presented.

### 2.1. Immunological Detection of Surface-Residing Molecular Targets

Immunological detection relies on the reaction between an antigen and an antibody. Quantitative visualization of the reaction takes place through the use of chromogenic substrates and the labeling of the antigen or antibody with enzymes that catalyze color development. In brief, the antigens or antibodies are immobilized within the wells of a microtiter plate, and a sample containing the respective antibodies or antigens is added and allowed to react. Then enzyme-conjugated secondary antibodies and the chromogenic substrate are added and the change in color intensity is measured. Although several types of Enzyme-Linked Immunosorbent Assays (ELISA) have been developed, the most common ones are indirect, sandwich and competitive ELISA [19]. Indirect ELISA is based on the capture of the antigen, which is immobilized on the adsorbent surface, by a primary antibody and the subsequent capture of the primary antibody with an enzyme-conjugated secondary antibody. On the other hand, in sandwich and competitive ELISA, the primary antibody, instead of the sample containing the antigen, is immobilized. In sandwich ELISA, the target microorganism is captured by the immobilized primary antibody and subsequently to another epitope by the enzyme-conjugated secondary antibody. Competitive ELISA differs from sandwich ELISA to the following: instead of enzyme-conjugated secondary antibody, an enzyme-conjugated antigen is applied and allowed to react with immobilized antibodies that have not captured an antigen from the sample. Therefore, color development is inversely correlated with the presence of the target antigen.

ELISA has been extensively applied and, therefore, has become a classical approach, especially in clinical practice. Detection is based on the specificity of antigen–antibody interaction, as well as the enzymatic reaction that signifies this interaction. It is an approach that is characterized by simplicity but suffers from limitations attributed to its structure. More specifically, cross-reactivity is the most frequently reported reason for false-positive results. Cross-reactivity refers not only to the capture of antigens not residing on the surface of the target microorganisms but also to the reaction between the primary and secondary antibodies. Although sandwich ELISA is characterized by improved sensitivity and specificity, compared to the other ELISA types, due to the capture of the target by two antibodies, the design and development of such an assay is rather demanding and the possible interactions between the antibodies limit its application [19]. In addition, the long and expensive preparation, the low stability of the antibodies, as well as constraints of the experimental procedure have been reported as the most pronounced pitfalls of this approach [20]. Some of the latter, especially the ones referring to the adsorbent surface and colorimetric detection, have been effectively addressed through the introduction of nanomaterials [21]. However, only marginal improvement has been achieved regarding the sensitivity and specificity of the approach, which was only feasible through the substitution of antibodies as recognition elements with aptamers, nanobodies or the use of haptens [22,23,24,25].

In Table 1, the application of ELISA-based detection of foodborne pathogens in real food samples is presented. In most of the cases, food samples were spiked with the strain(s) used for the development of each assay, leading to very promising results. In contrast, the assessment of naturally contaminating samples was only performed by Hadjilouka et al. [26] and Zhang et al. [27]. Such an assessment is much more challenging due to the occurrence of native microbiota and possible differences in the epitope used for capturing the cells of the pathogenic bacteria under examination, resulting from strain variability. The first was reported as the most probable reason for the large number of false positive results reported by Hadjilouka et al. [26], while assessing the prevalence of *L. monocytogenes* and *E. coli* O157:H7 in naturally contaminated cucumber samples. In contrast, Zhang et al. [27] reported that while assessing *E. coli* O157:H7 prevalence in various foodstuffs, the results obtained by double antibody sandwich ELISA were in accordance with the duplex-PCR method that was employed in parallel.

ELISA-based detection has been employed in a variety of formats, mostly immunofluorescence and lateral flow immunochromatography, as well as in the development of immunosensors [33,34,35,36,37,38,39]. In all cases, the attractive features are ease of use, portability and speed of analysis. The latter is compromised by the food matrix and the accompanying microbiota, the removal of which demands sample preparation and selective enrichment steps. This challenge has yet to be effectively addressed.

### 2.2. Use of Aptamers for the Detection of Surface-Residing Molecular Targets

Aptamers are short (<100-mer), single-stranded oligonucleotides (DNA or RNA), which may fold into three-dimensional structures that enable them to bind to specific targets. More specifically, the size, shape and charge of specific sites of these structures makes them complementary to various targets, the size of which may range from low molecular weight molecules to whole cells [40]. This binding may also result in regulatory functions (riboswitches) or the catalysis of specific biochemical reactions (ribozymes, DNAzymes) [41]. The binding capacity of specific aptamers, combined with the catalytic capacity of others, led to the development of aptazymes, namely synthetic nucleic acid molecules consisting of a binding and a catalytic domain [42].

Aptamers have been employed in biosensing and biomedical applications [43,44]. Regarding the latter, their use in medical imaging, diagnosis and treatment of diseases, targeted drug delivery, and regenerative medicine have already been described [44,45,46,47,48]. As far as biosensing applications are concerned, aptamers are used as recognition and binding elements. A series of technical and technological advantages, such as their ease of production, long shelf life, and stability over a wide range of temperatures and pH values, have led to a trend towards the substitution of antibodies in relevant protocols. In addition, the tunability of their properties, including size, chemical composition and binding affinity, resulted in the re-design of a number of antibody-based assays, such as ELISA, Western blotting, antibody arrays and immuno-PCR and the substitution of the antibodies with aptamers [49,50,51,52,53,54,55].

Aptamers have been extensively assessed as recognition elements of foodborne pathogens. Indeed, a series of oligonucleotides have been reported for selective capturing of pathogenic bacteria such as *Salmonella* serovars [56,57], *L. monocytogenes* [58,59], *E. coli* O157 [60,61], *Staphylococcus aureus* [62,63], *C. jejuni* [64], *Vibrio parahaemolyticus* [65], *V. vulnificus* [66] and *Shigella flexneri* [67]. The procedure used for the selection of the most suitable aptamers is termed the systematic evolution of ligands by exponential enrichment (SELEX). It consists of three steps: i. challenge of the aptamer to bind to a specific target under defined conditions; ii. removal of unbound aptamers; iii. amplification by PCR of the bound ones. Since in the majority of cases the starting point is a pool of randomly synthesized aptamers, after a number of cycles, the ones that bind more efficiently will be amplified [68,69,70].

Aptamers may also be designed in silico [71]. Indeed, knowledge of the aptamer sequence allows for the prediction of secondary and tertiary structures. Then, knowledge of the target structure allows the simulation of the aptamer–target interaction through docking analysis. Thus, through comparative analysis, the selection of aptamers that may lead to improved sensitivity and specificity of detection is facilitated.

Visualization of the complex formed by the aptamer and the target cells is a very critical issue. It has been achieved by many approaches, resulting in the development of a wide range of aptasensors. In brief, the aptamers may be conjugated with many different molecules, such as gold nanoparticles that enable colorimetric detection, carbon dots, quantum dots or 6-carboxyfluorescein (6-FAM) that are used for detection based on fluorescence. Detection may also be performed by measuring the changes in electrical properties or mass that take place upon capturing the target bacteria [72]. Specific attention should also be given to the effect that food constituents or the food matrix may have on detection and the need for additional analytical steps.

In Table 2, the application of aptamer-based detection of foodborne pathogens in actual food samples is presented. In the majority of cases, the pathogen is spiked into an extract of the commodity, obtained after dilution and homogenization. Such an approach may either indicate experimental steps that should be undertaken for effective application of the assay or that the focus lies on facilitating the visualization step rather than improving the conditions of detection in naturally contaminated samples, which are rarely included in the study. Thus, the majority of the literature highlights the effectiveness of aptamers in the detection of foodborne pathogenic bacteria but only under experimental conditions. Only in the study by Appaturi et al. [73] was the efficacy of the developed assay challenged in naturally contaminated samples, in parallel with established classical microbiological techniques for verification purposes. In that study, the results obtained by both approaches were in agreement. Therefore, further studies are necessary in order to exploit the whole potential of aptamers and, on the other hand, reveal and address practical constraints. Finally, another issue that deserves attention is the specificity of detection. This is usually assessed by challenging the aptamers with other foodborne pathogens or spoilage-associated microorganisms. Although such a study provides a good indication, specificity assessment should also take into consideration the native microbiota of the commodity under examination.

### 2.3. Use of Lectins for the Detection of Surface-Residing Molecular Targets

Lectins are bivalent or polyvalent proteins, present in nearly any living organism, with a unique carbohydrate-binding capacity, which may take place through hydrophobic interactions, hydrogen bonding, van der Waals interactions and metal ion coordination [74]. Currently, there are three lectin classification schemes based on their molecular structure, glucoconjugate specificity and source [75]. In general, each lectin exhibits carbohydrate specificity, on the basis of their unique amino acid sequence and concomitantly secondary and tertiary structures; for example, concanavalin A (a lectin from *Canavalia ensiformis*) and the agglutinins from *Lens culinaris* and *Pisum sativum* bind to a-D-mannose and α-D-glucose, the agglutinins from *Datura stramonium* and wheat germ to β-D-N-acetylglucosamine and the agglutinins for soybean and *Dolichos biflorus* to α-D-N-acetylglucosamine [76]. The significance of this carbohydrate-binding capacity has already been exploited for medical applications [77,78], while some efforts have been made regarding food safety monitoring [79]. Especially regarding the latter, capturing foodborne pathogens by lectins relies on the recognition of carbohydrate moieties in the bacterial cell envelope. More specifically, the D-glucose or N-acetylglucosamine residues found in some forms of the teichoic acids of Gram-positive bacteria, as well as similar residues of the lipopolysaccharide layer, such as the O-polysaccharides of Gram-negative bacteria, serve as binding sites of lectins [80].

The capacity of several lectins to react with foodborne pathogens has been studied to some extent. The results indicate that binding is strain specific and cross-reactivity should be expected. Indeed, Facinelli et al. [81] examined the reactivity of lectins from *Triticum vulgaris*, *Griffonia simplicifolia*, *Ricinus communis* and *Helix pomatia* with 46 *L. monocytogenes* and 3 *L. innocua* strains and verified the strain-specific nature of the binding. Interestingly, a correlation with virulent capacity was made since greater reactivity was detected among the virulent than the avirulent strains. The lectins from *T. vulgaris* and *R. communis* were reported to react with more *L. monocytogenes* strains than those from *G. simplicifolia* and *H. pomatia*. The very good reactivity of the lectins from *T. vulgaris* with *L. monocytogenes* was also verified by Raghu et al. [82]. Very good reactivity was also reported for the lectins from *H. pomatia*, opposing the results presented by Facinelli et al. [81].

**Table 2:** Studies describing the application of aptamer-based detection of foodborne pathogens in actual food samples.

Pathogen	Commodity	Comment	Reference
*V. parahaemolyticus*, *S*. Typhimurium	shrimp, chicken meat	The development of a dual FRET-based aptamer assay using amorphous carbon nanoparticles as fluorescence quencher and green-emitting quantum dots and red-emitting quantum dots as beacons. A filtrate of frozen fresh shrimps and chicken breast, which was prepared by 10 times dilution and homogenization of the samples with alkaline peptone containing 3% NaCl and PBS, respectively, was inoculated with the pathogens with population ≥10^3^ CFU/mL which were subsequently effectively detected. *E. coli*, *L. monocytogenes*, *Sh. dysenteriae* and *St. aureus* did not interfere with the analysis.	[83]
*S*. Typhimurium	apple juice	The development of a label-free impedimetric biosensor was reported. Apple juice was spiked with 10^2^–10^6^ CFU/mL of the pathogen, which was subsequently detected. Specificity was tested against *E. coli*, *K. pneumoniae*, *Eb. aerogenes* and *Ci. freundii* and did not interfere with the analysis.	[84]
*S*. Typhimurium	milk	The development of a luminescent bioassay employing gold nanorods as luminescence quencher and Mn^2+^-doped NaYF4:Yb,Tm upconversion nanoparticles as donor, was reported. Twenty times diluted, decreamed and filtered milk was spiked with the pathogen with population ≥10^3^ CFU/mL, which were subsequently effectively detected. Specificity of the aptamers was tested against *E. coli* and *St. aureus* did not interfere with the analysis.	[85]
*E. coli* O78:K80:H11	water, milk, guava, litchi and mango juices	An aptasensor for label-free impedimetric sensing of the pathogen was developed and effectively applied to detect the spiked strain down to 10 CFU/mL. *B. subtilis*, *Ci. braakii*, *E. coli* DH5α, *Eb. aerogenes*, *L. monocytogenes*, *Pr. vulgaris*, *Sh. boydii* and *Sh. flexneri* and did not interfere with detection.	[86]
*E. coli* O157:H7	ground beef	Ten times diluted and homogenized with PBS ground beef was spiked with the pathogen. Detection took place through a paper-based optical aptasensor to a detection limit of 233 CFU/mL. *E. coli* non-O157:H7, *L. monocytogenes*, *S*. Typhimurium and *St. aureus* did not interfere with the analysis.	[87]
*E. coli* O157:H7	ground beef	Ten times diluted and homogenized with PBS ground beef was spiked with the pathogen. Aptamers were conjugated to 4-aminothiophenol-gold nanoparticles that enabled detection of the pathogen through SERS analysis to a detection limit of 10^2^ CFU/mL. *E. coli* non-O157:H7, *L. monocytogenes*, *S*. Typhimurium and *St. aureus* did not interfere with the analysis.	[88]
*E. coli* O157:H7	milk	Milk samples were diluted 20 times and spiked with pathogen population ≥1.6 × 10^2^ CFU/mL. A colorimetric protocol was developed through the synthesis of copper-based metal-organic framework nanoparticles functionalized with aptamers that enabled the visual detection of the pathogen. *E. coli* non-O157:H7, *S*. Typhimurium, *St. aureus* and *L. monocytogenes* did not interfere with the detection.	[89]
*Salmonella*	chicken meat	An electrochemical aptasensor was developed that could detect *Salmonella* (serotypes Typhimurium, Albany, Enteritidis, Weltevreden, Typhi and Derby). *E. coli*, *Ec. faecalis*, *K. pneumoniae*, *P. aeruginosa* and *St. aureus* did not interfere with the analysis. Five samples were 10-fold diluted with BPW and incubated for 3 h at 37 °C. Three samples were found positive in *Salmonella* presence, with populations ranging between 10 and 10^3^ CFU/mL, which was verified by the culture-based method.	[73]
*S*. Paratyphi A	meat, chicken meat, milk	The development of a FRET-based aptamer assay having graphene oxide as fluorescence quencher and quantum dots as molecular beacon, was reported. PBS extract of the meat samples and 10-times diluted milk were inoculated with the pathogen with population ≥10^3^ CFU/mL, which were subsequently effectively detected. *E. coli*, *K. pneumoniae*, *P. aeruginosa*, *Sh. flexneri* and *St. aureus* did not interfere with the analysis.	[90]
*L. monocytogenes*	lettuce	An ELARCA assay was developed. The lettuce sample was spiked with 61–6.1 × 10^7^ CFU/g of the pathogen and 10 times diluted. Detection was performed in the precipitate. The LOD was calculated at 6.1 × 10^3^ CFU/g. Specificity was tested against *B. cereus*, *Cr. Sakazakii*, *S*. Enteritidis, *St. aureus*, *E. coli* O157:H7 and *P. aeruginosa*, which did not interfere with the analysis.	[91]
*L. monocytogenes*	milk	The development of a fluorescence aptasensor consisting of aptamer-functionalized upconversion nanoparticles to provide fluorescent signals and aptamer-functionalized magnetic nanoparticles for concentration of the complex with the pathogen. Milk was spiked with 10^2^–10^4^ CFU/mL of the pathogen and subsequently effectively detected. Detection was performed in the precipitate that was resuspended in PBS buffer. Specificity was tested against *E. coli* O157:H7, *S*. Typhimurium and *St. aureus*, which did not interfere with the analysis.	[92]
*S*. Typhimurium, *St. aureus*	milk	The development of an aptamer-based gold/silver nanodimer SERS probes for the simultaneous detection of *S*. Typhimurium and *St. aureus*, was reported. Milk was decreamed, filtered and diluted 20 times before being spiked with pathogen populations ≥10^2^ CFU/mL. The population detected was also verified by the classical microbiological technique. The specificity of the aptamers was tested against *E. coli*, *L. monocytogenes*, *Sh. dysenteriae*, *S*. Enteritidis, *S*. Paratyphi B, *St. epidermidis* and *St. saprophyticus*, which did not interfere with the analysis.	[93]
*E. coli*	coconut water, litchi juice, bread	Ten times diluted coconut water and litchi juice, as well as diluted and homogenized bread were spiked with the pathogen. The detection protocol used aptamers conjugated to Au nanoparticles and enclosed in graphene oxide, which enabled colorimetric detection via the naked eye. Visual detection of 10 cells/mL in the bread and coconut samples and 10^3^ cells/mL in the litchi juice sample were reported. *K. pneumoniae*, *Pr. vulgaris*, *Pr. mirabilis*, *Eb. aerogenes*, *St. aureus* and *P. aeruginosa* did not interfere with the detection.	[94]
*S*. Typhi	milk, egg	An electrochemical biosensor was developed for specific detection of *S*. Typhi. *S*. Typhimurium, *S*. Cotham, *E. coli* O157 and *Sh. sonnei* did not interfere with the analysis. Raw milk and eggs were homogenized and spiked with 2.1 × 10^5^ CFU/mL of the pathogen, which was detected by the aptasensor.	[95]
*L. monocytogenes*	pork meat, milk	The conjugates of aptamer-Fe_3_O_4_@ZIF-8, anti-*L. monocytogenes* antibody-biotin, streptavidin-FITC were employed for *L. monocytogenes* capture, signal amplification and fluorescence recognition, respectively. The supernatant of ten times diluted and homogenized pork meat or milk samples were spiked with 6.6 × 10^2^–6.6 × 10^4^ and 2.6 × 10^2^–2.6 × 10^4^ CFU/mL respectively, which were subsequently effectively detected. Specificity was tested against *E. coli* O157:H7, *S*. Typhimurium, *St. aureus*, *V. parahaemolyticus* and *P. aeruginosa*, which did not interfere with the analysis.	[96]

BPW: buffered peptone water; ELARCA: enzyme-linked aptasensor with rolling circle amplification; FRET: fluores-cence resonance energy transfer; LOD: limit of the detection; PBS: phosphate buffered saline; SERS: surface enhanced Raman spectroscopy; *B*.: *Bacillus*; *Ci*.: *Citrobacter*; *Cr*.: *Cronobacter*; *E*.: *Escherichia*; *Eb*.: *Enterobacter*; *Ec*.: *Enterococcus*; *K*.: *Klebsiella*; *L*.: *Listeria*; *P*.: *Pseudomonas*; *Pr*.: *Proteus*; *S*.: *Salmonella*; *Sh*.: *Shigella*; *St*.: *Staphylococcus*; *V*.: *Vibrio*.

On the contrary, the respective from *Phytolacca americana*, *Maackia amurensis* and *Pisum sativum* exhibited the least reactivity. The lectins from *T. vulgaris* were only marginally reactive with *L. ivanovii*, *L. innocua*, *B. cereus*, *St. aureus*, *Lactococcus lactis*, *Limosilactobacillus fermentum* and *Leuconostoc mesenteroides* but presented noticeable reactivity with *S*. Choleraesuis, *K. pneumoniae* and *Ci. freundii* strains that were examined. The capacity of the lectins from *T. vulgaris* to bind to *L. monocytogenes*, *St. aureus*, *Salmonella* spp. and *E. coli* was also reported by Payne et al. [97]. In the same study, the capacity of lectins from *Agaricus bisporus* to bind primarily to *L. monocytogenes* and *St. aureus* and only marginally to *Salmonella* spp., as well as the capacity of lectins from *H. pomatia* to bind to *St. aureus* and *L. monocytogenes*, were also reported. This is also the case for lectins from marine organisms. Indeed, lectins from a series of algae, sponges, mollusks, arthropods, echinoderms and fishes have been reported to bind to a variety of prokaryotic and eukaryotic microorganisms [98].

Lectins have also been employed as biorecognition elements for the detection of foodborne pathogens [99,100]. Despite their advantages, namely their ubiquitous nature, low cost and high stability, their application is still limited, mostly due to the extensive cross-reactivity.

## 3. Metabolites as Molecular Targets

The presence of pathogenic bacteria, as well as the quantification of their population, has also been assessed through qualitative and quantitative detection of the volatile compounds that they produce. Thus, the occurrence of several compounds, including aromatic, hydrocarbons, ketones, fatty acids, alcohols, sulfur- and nitrogen-containing ones, has been correlated with the metabolic activities of several pathogenic bacteria, such as *E. coli*, *S*. Typhimurium, *L. monocytogenes*, *St. aureus* and *Sh. sonnei* in chemically defined media [101,102,103,104,105].

The detection and differentiation of pathogenic bacteria, on the basis of the response of the sensors used in electronic nose devices, has been proposed by many authors. This approach, which is applicable in in vitro systems, includes growth of the bacteria under defined conditions, use of a commercially available or customized electronic nose device, followed by clustering and differentiation by statistical methods [106,107,108].

A more sophisticated approach was developed through the exogenous addition of substrates that target enzymatic activities specific to the desired taxonomic or epidemiologic level of the detection. Such enzymatic activities were designed to liberate VOCs, the detection of which has been attempted by a number of techniques. This approach seems to be largely affected by substrate concentration, as well as incubation temperature and time. This strategy has been employed for the detection of several foodborne pathogens. More specifically, *E. coli* produces 2-nitrophenol and 4-nitrophenol from 2-nitrophenyl-β-D-galactoside and 4-nitrophenyl-β-D-glucuronide through the β-galactosidase and β-D-glucuronidase activities, respectively [109,110]. *Pseudomonas aeruginosa* produces alanine upon the addition of 3-amino-N-phenylpropanamide, TFA salt through β-alanine aminopeptidase activity. *Klebsiella pneumoniae* and *Enterococcus faecium* 2-nitrophenol from 2-nitrophenyl-β-D-glucoside via β-glucosidase activity [111]. Tait et al. [112] reported the detection of *L. monocytogenes* on the basis of detection of 2-nitrophenol and 3-fluoroaniline, which were liberated by the activities of β-glucosidase and hippuricase upon exogenous addition of 2-nitrophenyl-b-D-glucoside and 2-[(3-fluorophenyl) carbamoylamino]acetic acid, respectively. Taylor et al. [113] reported the differentiation between *L. monocytogenes* and non-pathogenic listeriae through the utilization of two enzyme substrates, namely benzyl-α-D-mannopyranoside and D-alanyl-3-fluoroanilide. The former liberates benzyl alcohol in the presence of α-mannosidase activity and the latter liberates 3-fluoroaniline in the presence of D-alanine aminopeptidase activity. Usually, *L. monocytogenes* strains exhibit α-mannosidase activity but do not possess D-alanine aminopeptidase. In contrast, D-alanine aminopeptidase is produced by nonpathogenic listeriae, which may also possess α-mannosidase [113].

The aforementioned approaches have been developed for the in vitro differentiation of foodborne pathogens. However, when detection is attempted in food samples, the occurrence of native microbiota complicates this analysis. This interference could be the reason for the poor accuracy of *S*. Typhimurium detection in beef meat stored at 4 and 10 °C for up to 7 days reported by Balasubramanian et al. [114]. To address this issue and improve the reliability of detection, the implementation of additional steps, such as selective enrichment, is necessary. Tait et al. [112], while attempting to develop a protocol for *L. monocytogenes* detection in milk, reported interference by non-pathogenic listeriae, as well as microorganisms characterized as part of the native milk microbiota. The detection of the pathogen was based on the liberation of 2-nitrophenol and 3-fluoroaniline through the activities of β-glucosidase and hippuricase targeted through the exogenous addition of 2-nitrophenyl-b-D-glucoside and 2-[(3-fluorophenyl) carbamoylamino]acetic acid, respectively. Non-pathogenic listeriae, as well as *Ec. faecium*, *Ec. faecalis* and *Lactobacillus acidophilus* also exhibited these enzymatic activities, even after the selective enrichment procedures that were examined. An interference to the detection of *S*. Stanley by native milk microbiota was reported by Bahroun et al. [115]. *Salmonella* detection was based on the release of 2-chlorophenol and phenol liberated by the activities of C8 esterase and a-galactosidase targeted through the exogenous addition of 2-chlorophenyl octanoate and phenyl a-D-galactopyranoside. At the same time, L-pyrrollidonyl fluoroanilide was also added, targeting pyrrolidonyl peptidase activity through the release of 3-fluoraniline, which is not present in *Salmonella*. The authors reported that interference by native microbiota was avoided through the proposed optimized enrichment procedure.

In Table 3, studies assessing the presence of pathogenic bacteria in food samples through the detection of their volatile compounds are presented. In general, the capacity of this approach has been exhibited, either through the use of electronic noses or through the detection of specific volatile compounds. In the majority of the studies, the aim was to distinguish uninoculated from inoculated samples, without any attempt to detect the pathogen under consideration in naturally contaminated samples and compare the performance of this approach with the respective established ones (e.g., ISO protocols). Therefore, further study is still necessary in order to elucidate whether this approach is suitable to be considered as complementary or even substitute the established ones.

## 4. Cellular Components as Molecular Targets

The cellular components that may provide the necessary discrimination capacity are nucleic acids. Through the assessment of appropriate nucleotide sequences, the desired taxonomic depth, even the strain level, can be reached. Therefore, nucleic acids have been extensively considered as molecular targets for the detection and quantification of foodborne pathogens. Apart from the limitations mentioned in the introduction, the employment of such an approach necessitates the inclusion of a pretreatment step that may enable differentiation between living and dead or dormant cells. Indeed, DNA may persist in the microenvironment after cell death; therefore, its use may reveal the microbiological history of a sample rather than the microbiological quality at a specific time [125]. The exclusion of environmental DNA or DNA of cells with compromised cell membranes from the subsequent analytical steps has been achieved through the use of propidium monoazide (PMA) [126]. However, the use of PMA inserts limitations that affect the quality of the detection and therefore need to be effectively addressed. Indeed, the quality of the detection is largely affected by the amplicon size of the subsequent PCR step, as well as the population of the target pathogen and the dead-to-viable ratio. The definite exclusion of environmental DNA from the subsequent PCR has been reported when the generated amplicon exceeds 1000 bp [127,128,129]. This issue was also effectively addressed with the use of platinum and palladium compounds that chelate DNA [130,131]. As far as the pathogen population and the dead-to-viable ratio were concerned, Pan and Breidt [132] reported that a minimum population of 10^3^ CFU/mL, and a ratio of dead to viable cells below 10^4^ CFU/mL are necessary for effective detection. Finally, PMA may also penetrate living cells when the cell density is too high [133,134]. Cell viability can alternatively be assessed through the use of thiazole orange monoazide (TOMA) [135] or DyeTox13 Green C-2 Azide [136]. Both dyes detect metabolically active cells instead of cells with compromised cell membranes, offering improved accuracy. However, it seems that the aforementioned limitations referring to the amplicon size resulting from the subsequent detection step still remain.

The use of RNA, particularly rRNA, has been proposed as an alternative to DNA because of its better correlation with living cells [137,138,139]. In this case, a reverse transcription reaction should precede the PCR. Microecosystems assessment through the comparative utilization of DNA and RNA have revealed that, in some cases, the utilization of RNA may reveal more information [125,138,140].

The utilization of phages may also assist in the discrimination between dead and living or viable but non-culturable bacteria [141]. Indeed, a variety of methods have been developed on the basis of the interactions between phages and host cells [142]. Detection of these interactions is usually achieved through genetic modification of the phage to overexpress β-galactosidase, alkaline phosphatase or the *lux* gene, coupled with subsequent colorimetric detection, in solid or liquid media [143,144,145,146]. However, its application in food is limited by the interference of food matrix constituents in signal detection [147].

A wide variety of nucleic acid-based approaches have been developed for the detection of foodborne pathogens. They are based on the detection of virulence-associated genes or segments of the CRISPR-cas system through the use of suitable primers, amplification (in the majority of cases) of the respective DNA segment, and visualization of the amplicon. As already mentioned, this procedure is compromised by the occurrence of background microbiota, the population of which may exceed the respective of the target bacteria, as well as the possible occurrence of inhibitors of the amplification process, which takes place mostly through PCR. In the first case, non-specific amplification may lead to false positive results, while in the second case, inhibition of the reaction may generate false negative ones. The most effective ways to prevent false positive results are by adjusting PCR conditions to increase specificity (e.g., reduction of Mg^2+^, increase of annealing temperature, etc.) or by using hybridization probes. In addition, verification of annealing specificity can be performed by amplicon sequence assessment by, e.g., sequencing reaction or melting curve analysis. The usefulness of the latter is getting increased recognition, and its ability to reach strain level has been exhibited [148]. Therefore, RT-qPCR protocols combining the aforementioned adjustments are the method of choice for the detection and quantification of foodborne pathogenic bacteria in food samples in many studies [149,150,151,152]. By combining the utilization of RNA as the target molecule with suitably adjusted PCR conditions and the use of hybridization probes or melting curve analysis, the detection and quantification of metabolically active bacterial foodborne pathogens with adequate sensitivity can be achieved. On the other hand, the most effective way to prevent false negative results is the droplet digital approach [153]. Indeed, the subdivision of the sample DNA in droplets and the endpoint assessment make it less sensitive to PCR inhibitors, at least compared to the quantification alternative, namely qPCR [154]; therefore, it has also been employed for foodborne pathogenic bacteria detection and quantification [154,155,156,157,158]. However, neither approach is suitable for routine analysis due to the high cost of the equipment and consumables and the need for experienced personnel for RNA handling and effective protocol execution.

All the above approaches require the use of laboratory equipment and several experimental steps. In addition, these requirements limit the incorporation of PCR into biosensors. Isothermal techniques were developed to address this issue and allowed the development of autonomous and portable systems [159]. Several approaches for the isothermal amplification of DNA have been developed [160]. The ones that are more frequently present in the literature are nucleic acid sequence-based amplification (NASBA) [161], loop-mediated isothermal amplification (LAMP) [162], rolling circle amplification (RCA) [163], recombinase polymerase amplification (RPA) [164], helicase dependent amplification (HDA) [165], sequence exchange amplification (SEA) [166] and recombinase-aided amplification (RAA) [167]. Regarding their application in the detection of foodborne pathogens in food samples, LAMP is the most frequently employed technique.

In Table 4, recent studies that use nucleic acids as a molecular target for the detection of foodborne pathogens in food samples are exhibited.

Given the large number of similar studies, the aim of the table was to highlight the diversity of the available approaches and emphasize the capacity for combination between the available strategies for capturing the target sequences, signal generation and detection. Early attempts included the use of PCR, in simplex or multiplex format, for DNA isolated from the enrichment broths used for classical microbiological assessment. Due to the susceptibility of this approach to false positive and false negative results, for the reasons already explained, such an approach could only be used with extreme caution and only as an indication of the presence or absence of foodborne pathogens in food samples. The use of quantitative PCR improved the time required for detection, as well as the ability to reduce or detect false positive results, through the use of hybridization probes and melting curve analysis, respectively. However, such an approach increased the cost of detection and required trained personnel [176]. Attempts to concentrate the target cells (e.g., through immunological techniques) have not improved the sensitivity and specificity of the detection and, therefore, have not met wide acceptance [177].

Currently, research has focused on the utilization of more sophisticated approaches, such as isothermal amplification and next-generation sequencing (NGS). Regarding the first, LAMP is the one most extensively employed. Despite the high practical significance of this approach, its application is limited by the rather complicated nature of primer design and its susceptibility to false positive results [178,179]. Similarly, NGS approaches have been increasingly used in studies on the characterization of microecosystems. Despite their increased usefulness, their application in foodborne pathogenic bacteria detection is limited by the lack of standardized workflows that provide consistency and the inferior sensitivity compared to classical microbiological techniques [180,181].

## 5. Conclusions

The discovery and evaluation of suitable molecular markers for the effective detection of foodborne pathogens have been extensively assessed. Several such markers, along with their detection methods, have been proposed and have their endogenous advantages and limitations experimentally verified. The greatest challenges are imposed by the food composition and matrix and the accompanying microbiota. Based on the availability of physicochemical and bioinformatic tools and procedures and the cumulative advantages of their combination, it seems reasonable to expect that the classical microbiological approaches used for the detection of foodborne pathogens will eventually be replaced by procedures based on the detection of molecular markers.

## Figures and Tables

**Table 1:** Studies describing the application of ELISA-based detection of foodborne pathogens in actual food samples.

Pathogen	Commodity	Comment	Reference
*E. coli* O157:H7, *L. monocytogenes*	cucumber	The monoclonal anti-*E. coli* O157 (ab20976) and the monoclonal anti-*L. monocytogenes* (ab11439) were employed for pathogen capture and the polyclonal secondary antibody (ab47827) for visualization. Cucumber peels were spiked with *E. coli* O157:H7 and *L. monocytogenes* at populations ranging from 0.9 to 6.9 log CFU/g and from 0.9 to 5.9 log CFU/g, respectively. Samples were lyophilized and further treated for indirect ELISA. A LOD of less than 3 log CFU/g was reported.	[28]
*L. monocytogenes*	milk	The development of an asymmetrically anchored cantilever sensor for the detection of *L. monocytogenes* was reported. The protocol was able to detect 10^3^ cells/mL in a single binding step. The addition of a secondary antibody step reduced false positive results, while the detection limit was reduced to 10^2^ cells/mL through the incorporation of a third antibody binding step.	[29]
*E. coli* O157:H7, *L. monocytogenes*	cucumber	The indirect ELISA method developed by Cavaiuolo et al. [28] was employed. The detection of *E. coli* O157:H7 and *L. monocytogenes* in naturally contaminated cucumbers was also performed by classical microbiological methods. Indirect ELISA was performed without prior and after enrichment steps. Extended cross reactivity resulted in a high number of false positive results.	[26]
*L. monocytogenes*	various foods	Novel specific antibodies were developed and screened with *L. monocytogenes* as target. Then, a bead array for the detection of *L. monocytogenes* was developed and the efficacy of the detection was examined in a series of spiked foods (spinach, bean sprout, potato, lettuce, melon, egg, chicken beef, pork, whole milk, skimmed milk). The LOD ranged between 10^4^–10^5^ CFU/mL with the 3C3 antibody. LOD could be reduced when selective enrichment was employed. Already developed antibodies for the detection of *Salmonella* (ab8273) and *Campylobacter* (C818) were combined with the anti-*Listeria* ones to enable pathogen detection in a multiplex format. Capturing of *C. jejuni* by *Salmonella* antibodies was reported.	[30]
*Salmonella*	milk	Novel monoclonal antibodies against *Salmonella* core lipopolysaccharide were obtained. Then, the development of a cross-reactive sandwich ELISA for *Salmonella* spp. (serotypes Paratyphi A, Typhimurium, Thompson, Enteritidis, Anatum, Arizona) was reported. The LOD ranged from 1.56 × 10^6^ to 1.25 × 10^7^ CFU/mL. Milk was spiked with 1 CFU/mL, which was detected after 24 h enrichment.	[31]
*E. coli* O157:H7	various foods	Novel monoclonal and polyclonal antibodies against *E. coli* O157:H7 intimin gamma 1 were generated and a double antibody sandwich ELISA protocol was developed. *S*. Enteritidis, *L. monocytogenes*, *Sh. flexneri*, *Str. suis* and a variety of *E. coli* serotypes did not interfere with the analysis. A total of 498 field samples, including 300 food samples, were analyzed by the ELISA protocol developed and by duplex PCR, providing comparable results.	[27]
*S*. Enteritidis	milk	A nanobody library was built and screened against *S*. Enteritidis. Then, a double nanobody-based sandwich ELISA for the detection of *S*. Enteritidis was developed. Milk samples were spiked with ≥10^6^ CFU/mL, which were effectively detected. The LOD was reduced to 10 CFU/mL after selective enrichment.	[32]
*S*. Typhimurium	juice, honey, milk, pork	Phage-displayed nanobodies were generated and a double-nanobody sandwich immunoassay for the detection of *S*. Typhimurium was developed. The food samples were diluted with PBS, centrifuged and the supernatant was spiked with <10 cells of the pathogen. Effective detection took place after 6–8 h of selective enrichment.	[20]

LOD: limit of detection; *C*.: *Campylobacter*; *E*.: *Escherichia*; *L*.: *Listeria*; *S*.: *Salmonella*; *Sh*.: *Shigella*; *Str*.: *Streptococcus*.

**Table 3:** Studies assessing the presence of pathogenic bacteria in food samples through the detection of their volatile compounds.

Pathogen	Commodity	Detection Methodology	Comment	Reference
*E coli*	alfalfa (*M. sativa* L.) sprouts	EN (Fox 3000)	Alfalfa (*M. sativa* L.) sprouts were spiked with 10^5^ CFU/g *E. coli* and stored at 10 °C for up to 3 d. Inoculated and uninoculated samples were effectively differentiated by the electronic nose. Prediction of the population of the pathogen was attempted through an artificial neural network, exhibiting a good correlation between actual and predicted data.	[116]
*S*. Typhimurium	beef meat	EN (homemade)	Beef meat was spiked with 10^4^ CFU/mL *S*. Typhimurium and stored at 20 °C for up to 4 days. The authors proposed data analysis by a novel procedure termed Independent Component Analysis. The model developed on the independent components exhibited better performance and revealed more information than PCA.	[117]
*E. coli*	alfalfa (*M. sativa* L.) seeds	EN (Fox 3000)	Alfalfa (*M. sativa* L.) sprouts were spiked with 10^5^ CFU/g *E. coli* and stored at 10 °C for up to 3 d. The authors proposed a Kohonen self-organizing map algorithm for the effective classification of contaminated samples.	[101]
*E. coli*	canned tomatoes	DHS, GC-MS, EN (ESO835)	Canned tomatoes were spiked with 400 CFU/mL *E. coli* and stored at 37 °C for 7 d. o-methyl styrene, ethynyl benzene and ocimene were detected in the samples inoculated with *E. coli* but not detected in uninoculated samples. Based on the nature and relative abundance of the volatile compounds detected, as analyzed by GC-MS or EN, PCA managed to differentiate inoculated samples from uninoculated ones.	[118]
*E. coli*	goat meat	EN (Cyranose-320)	Goat meat was spiked with 7.5 log CFU per 2 × 3 cm meat piece *E. coli* at stored at room temperature for 2–4 h. The PCA applied could not accurately classify the contaminated samples.	[119]
*S.* Typhimurium	beef meat (packaged aged and fresh)	HS-SPME/GC-MS	Packaged aged and fresh beef was spiked with 10^3^–10^4^ CFU/g *S*. Typhimurium and stored at 20 °C for 4 d. The presence of 2-pentanone and 3-methyl-2-butanone only in uninoculated fresh and aged beef samples, respectively, and not in inoculated ones, was reported. The VCs whose concentration was reported to change significantly with *Salmonella* counts were 3-hydroxy-2-butanone in fresh beef and 3-methyl-1-butanol, 3-hydroxy-2-butanone, acetic acid and 2-butanone in aged beef.	[120]
*S*. Typhimurium	beef meat	EN (homemade) EN (cyranose 320)	Beef meat was spiked with *S*. Typhimurium and stored at 4 and 10 °C for up to 7 d. Signals from both systems were combined in order to improve accuracy. The accuracy of classification was above 80% for samples stored at 10 °C and relatively low for those stored at 4 °C.	[114]
*L. monocytogenes*	milk	HS-SPME/GC-MS	Milk was spiked with 1–1.5 × 10^0^ to 1–1.5 × 10^7^ CFU/mL *L. monocytogenes* and stored overnight at 37 °C. Detection was based on the liberation of 2-nitrophenol and 3-fluoroaniline through the activities of β-glucosidase and hippuricase targeted through the exogenous addition of 2-nitrophenyl-b-D-glucoside and 2-[(3-fluorophenyl) carbamoylamino]acetic acid, respectively. Optimized enrichment procedure, failed to avoid interference by *L. welshimeri*, *L. innocua*, *L. ivanovii*, *Ec. faecium*, *Ec. faecalis* and *Lb. acidophilus*.	[112]
*E. coli*	mixed vegetable soup	EN (EOS507C)	Mixed vegetable soup was spiked with 10–10^2^ CFU/100 mL product *E. coli* and stored at 35 °C up to 24 h. PCA analysis of the raw data obtained after 24 h of incubation as well as LDA classification, managed to differentiate inoculated from uninoculated ones.	[121]
*S.* Typhimurium	alfalfa (*M. sativa* L.) seeds	EN (fox 3000)	Alfalfa (*M. sativa* L.) seeds were spiked with 3, 4, 5 and 6 log CFU/g *S*. Typhimurium and stored at 10 °C for 48 h. PCA effectively differentiated samples inoculated with 4, 5 and 6 log CFU/g from the uninoculated ones. The Kohonen network allowed effective visualization and clearer separation of the different sample groups.	[122]
*S*. Stanley	milk	HS-SPME/GC-MS	Milk was spiked with 4 log CFU/mL *S*. Stanley and stored at 37 °C for 5 h. *Salmonella* detection was based on the detection of 2-chlorophenol, phenol and not 3-fluoraniline, liberated by the activities of C8 esterase, a-galactosidase and pyrrolidonyl peptidase, targeted through the exogenous addition of 2-chlorophenyl octanoate, phenyl a-D-galactopyranoside and L-pyrrollidonyl fluoroanilide, respectively. The optimized enrichment procedure, in order to avoid interference by the native microbiota of the sample, allowed effective detection of *Salmonella* after 5 h incubation at 37 °C.	[115]
*Salmonella* spp., *Shigella* spp., *Staphylococcus* spp.	apples (Royal Delicious)	EN (homemade prototype)	A tri-layer approach consisting of GC-MS data, bacterial counts and data classification was used to create a reference table that was included in the processor of the EN enabling real-time quality assessment.	[123]
*S*. Typhimurium	pork meat (fresh)	EN (PEN3)	Fresh pork meat was spiked with 2, 4, 7 log CFU/g *S*. Typhimurium and stored at 50 °C for 300 sec. Principal component analysis managed to successfully discriminate uninoculated samples from inoculated ones at different contaminant levels. Moreover, support vector machine regression with a metaheuristic framework using genetic algorithm, particle swarm optimization and grid searching optimization algorithms provided satisfactory quantification of the pathogen.	[124]

DHS: Dynamic headspace analysis; EN: Electronic nose; GC-MS: Gas chromatography–mass spectrometry; HS-SPME: headspace solid-phase microextraction; LDA: Linear Discriminant Analysis; PCA: principal component analysis; VCs: volatile compounds; *E*.: *Escherichia*; *Ec*.: *Enterococcus*; *L*.: *Listeria*; *Lb*.: *Lactobacillus*; *M*.: *Medicago*; *S*.: *Salmonella*.

**Table 4:** Studies describing the application of nucleic acid-based detection of foodborne pathogens in food samples.

Pathogen	Commodity	Comment	Reference
*St. aureus*	milk powder, meat	The development of a method combining PMA with qPCR for the detection of *St. aureus* based on the amplification of *nuc* gene, was reported. The method was evaluated in spiked milk powder and meat products. PMA assisted in the exclusion of dead cells from the detection step and the initial inoculum of 10^5^ CFU/g was effectively detected.	[168]
*S.* Typhimurium	apple juice	The application of a novel biosensor for the detection of *S*. Typhimurium through the detection of Det7 phage tail protein via SPR. The capacity of the biosensor was evaluated in spiked apple juice; *S*. Typhimurium population above 5 × 10^5^ CFU/mL yielded sufficient signals.	[147]
*L. monocytogenes*, *Salmonella* spp., *E. coli* O157	milk	The development of a multiplex colorimetric LAMP-based technique for the detection of *L. monocytogenes*, *Salmonella* sp. and *E. coli* O157 targeting *plcA*, *invA* and *rfbE*, respectively, was reported. Detection was possible after 7 h of enrichment. The LOD95 in spiked UHT, fresh and raw milk was calculated at 1.6 CFU/25 mL for *Salmonella* sp. and *E. coli* O157 and 79.0 CFU/25 mL for *L. monocytogenes*.	[169]
*V. parahaemolyticus*, *St. aureus*, *Salmonella* spp.	seafood	The development of a multiple fluorescent probe-based LAMP approach for the simultaneous detection of *V. parahaemolyticus*, *St. aureus* and *Salmonella* spp., based on the amplification of *toxR*, *nuc* and *fimY*, respectively, was reported. The feasibility of the technique was evaluated in spiked seafood samples, as well as in naturally contaminated ones. The LOD in spiked samples after 18 h of enrichment in BPW was calculated at 5 CFU/25 g. Naturally contaminated samples were analyzed in parallel with classical microbiological techniques; both approaches yielded the same results after 18 h of enrichment in BPW.	[170]
*K. pneumoniae*	PIF	A method based on the combination of RAA with TOMA dye for the detection of *K. pneumoniae* in PIF was developed. The LOD in spiked PIF was calculated at 2.3 × 10^4^ CFU/g and at 3 CFU/g after 3 h pre-enrichment.	[171]
*L. monocytogenes*, *Salmonella* spp., *St. aureus*	eggs	A sensor based on electrical resistance microsphere counter and DNA hybridization, without prior DNA amplification step, for the simultaneous detection of *L. monocytogenes*, *Salmonella* spp. and *St. aureus*, targeting *hly*, *spuB* and *nuc*, respectively, was developed. The sensor was evaluated in spiked egg samples. After 3 h enrichment, the LOD was calculated at 20, 89 and 94 CFU/mL for *L. monocytogenes*, *Salmonella* spp. and *St. aureus*, respectively.	[172]
*L. monocytogenes*	cheese	The combination of SEA with surface-enhanced Raman spectroscopy for the detection of *L. monocytogenes* was reported. Detection was based on the isothermal amplification of a hypervariable region of 16S rDNA and capturing of the amplicons by streptavidin-modified magnetic bead and Au^MB^@Ag-isothiocyanate fluorescein antibody. The effectiveness of the approach was evaluated in spiked cheese samples, and the detection of as low as 20 CFU/mL of the pathogen was obtained.	[173]
*E. coli* O157:H7 (three strains cocktail)	meat, vegetables and milk	Solid phase reversible immobilization beads were used to bind and therefore concentrate the DNA of the spiked strains. Detection was based on a high-resolution melting curve multiplex real-time PCR assay targeting *eaeA*, *stx1* and *stx2*. With this approach, detection of the 10 CFU/mL inoculum was achieved without an enrichment step in the case of chicken breast, packaged leafy greens and romaine lettuce, after 4 h enrichment in the case of ground beef, ground turkey, ground chicken, green bell pepper and tomato. Enrichment for 8 h was necessary for the detection of the pathogen in the spinach and milk samples. Surprisingly, the detection of the spiked pathogen on the green onion, even after 8 h of enrichment, could not be achieved.	[174]
*Salmonella* sp., *S.* Typhimurium, *S.* Enteritidis	duck, mutton, pork, chicken	A 3-plex droplet digital PCR assay for the detection of *Salmonella* sp., *S*. Typhimurium and *S*. Enteritidis was developed. The pathogens were detected in spiked lettuce, milk and chicken juice samples to an LOD of 10 CFU/mL in the first case and 10^2^ CFU/mL in the last two. Naturally contaminated duck, mutton, pork and chicken samples were also analyzed in parallel to classical microbiological techniques; the assay exhibited very good concordance.	[155]
*St. aureus* ST398	milk, beef, lettuce	An enhanced colorimetric platform based on CRISPR/Cas12a system and label-free DNA-AuNP probe was developed. The platform was used to effectively detect *St. aureus* ST398 spiked in milk, beef and lettuce samples to an LOD of 5.8 × 10^4^, 5.8 × 10^3^ and 5.8 × 10^3^ CFU/g, respectively. Detection was also performed in the naturally contaminated samples.	[175]

BPW: buffered peptone water; LAMP: loop-mediated isothermal amplification; LOD: limit of detection; PIF: powdered infant formula; PMA: propidium monoazide; RAA: recombinase-aided amplification; SEA: sequence exchange amplification; SPR: surface plasmon resonance; TOMA: thiazole orange monoazide; *E*.: *Escherichia*; *K*.: *Klebsiella*; *L*.: *Listeria*; *S*.: *Salmonella*; *St*.: *Staphylococcus*; *V*.: *Vibrio*.

## Data Availability

Not applicable.
